# Transplantation of Porcine Hepatocytes Cultured with Polylactic Acid-O-Carboxymethylated Chitosan Nanoparticles Promotes Liver Regeneration in Acute Liver Failure Rats

**DOI:** 10.1155/2011/797503

**Published:** 2011-03-30

**Authors:** Zhong Chen, Renan Chang, Weijun Guan, Hongyu Cai, Fei Tang, Wencai Zhu, Jiahui Chen

**Affiliations:** Department of Hepatobiliary Surgery, Affiliated Hospital, Nantong University, 20 Xisi Road, Nantong 226001, China

## Abstract

In this study, free porcine hepatocytes suspension (Group A), porcine hepatocytes embedded in collagen gel (Group B), porcine hepatocytes cultured with PLA-O-CMC nanoparticles and embedded in collagen gel (Group C), and PLA-O-CMC nanoparticles alone (Group D) were transplanted into peritoneal cavity of ALF rats, respectively. The result showed that plasma HGF levels were elevated post-transplantation with a peak at 12 hr. The rats in Group C showed highest plasma HGF levels at 2, 6, 12, 24 and 36 hr post-transplantation and lowest HGF level at 48 hr. Plasma VEGF levels were elevated at 48 hr post-transplantation with a peak at 72 hr. The rats in Group C showed highest plasma HGF levels at 48, 72, and 96 hr post-transplantation. The liver functions in Group C were recovered most rapidly. Compared with Group B, Group C had significant high liver Kiel 67 antigen labeling index (Ki-67 LI) at day 1 post-HTx (*P* < .05). Ki-67 LI in groups B and C was higher than that in groups A and D at days 5 and 7 post-HTx. In conclusion, intraperitoneal transplantation of porcine hepatocytes cultured with PLA-O-CMC nanoparticles and embedded in collagen gel can promote significantly liver regeneration in ALF rats.

## 1. Introduction

 Acute Liver Failure (ALF) is a life-threatening clinicopathological condition with a high rate of fatality. Impaired liver regeneration is one of the most critical issues in the prognosis. Because of the larger potential regeneration capacity of liver, temporary and effective liver function support will make the patients with ALF have time to wait for liver transplantation and have the possibility of recovery through the regeneration of remaining hepatocytes. Hepatocytes transplantation (HTx) is anticipated to be an effective method to substitute liver functions [1, 2]. But because of the shorter survival time of transplanted hepatocytes, emphasis in study of HTx has been put on getting the better functions of transplanted hepatocytes and the better liver regeneration of ALF recipients. Nanomaterial scaffold is helpful to adherence, proliferation, and differentiated functions of cells [3]. In our previous study, we found that hepatocytes could proliferate rapidly and represent better functions on nanomaterial scaffold [4]. In this study, porcine hepatocytes cultured with polylactic acid-O-carboxymethylated chitosan (PLA-O-CMC) nanoparticles and embedded in collagen gel were transplanted into the peritoneal cavity of ALF rats to study the liver regeneration reaction. 

Hepatocyte growth factor (HGF) is a multipotent growth factor that is a powerful stimulator of DNA synthesis in a variety of cell types, especially hepatocytes [5]. HGF plays a key role in the regulation of liver regeneration after hepatocyte damage. It was reported that HGF activity increases in proportion to the decrease in functional liver mass before the initiation of liver regeneration and rapidly decreases to near normal levels after recovery [6]. Changes in HGF production reflect the status of regeneration process. Vascular endothelial growth factor (VEGF) is a strongest factor in a number of known endogenous factors promoting vessel regeneration. VEGF derived from hepatocytes is associated with processes of liver regeneration. HTx has been used by many investigators to demonstrate metabolic support and improve survival in rats with ALF. However, few of these reports have examined the impact of cell therapy on the regenerative response in the native liver. To our knowledge, there was no detailed study about liver regeneration response in HTx with nanomaterials. In the present study, levels of HGF and VEGF, albumen (ALB), alanine aminotransferase (ALT), total bilirubin (TB), and NH_3_ in the plasma and liver Kiel 67 antigen labeling index (Ki-67 LI) of ALF rats after HTx were observed. The aim of the present study was to investigate the effect of transplantation of porcine hepatocytes cultured with PLA-O-CMC nanoparticles and embedded in collagen gel on liver regeneration in ALF rats.

## 2. Materials and Methods

### 2.1. Animals

Chinese experimental miniature pigs (*n* = 5, male and female, body weight 2 to 4 Kg), Sprague-Dawley rats (*n* = 220, male and female, weight 250 to 280 g) were supplied by Experimental Animal Center of Nantong University. All operations were performed between 9 AM and noon. The pigs and rats were allowed free access to water and were fasted for 12 hr before experiment. The research protocol was in compliance with Chinese guidelines for the humane care of experimental animals. The study was approved by the hospital ethics committee.

### 2.2. Reagents

D-galactosamine (D-Gal) was purchased from the Department of Chemistry, Chongqing Medical University, China. Collagenase IV, RPMI1640, new-born bovine serum (NBS), and HGF were purchased from Gibco BRL, Life Technologies, USA. Polylactic acid was from Sigma Chemical Co., USA (St. Louis, MO). O-carboxymethylated chitosan was from Weikang Biotechnology Company Limited, Shanghai, China.

### 2.3. Preparation of PLA-O-CMC Nanoparticles

PLA-O-CMC nanoparticles were prepared with polylactic acid and O-carboxymethylated chitosan by u1trasonic method as described previously [7].

### 2.4. ALF Rat Model and Groups

10% D-Gal was injected into the peritoneal cavity of Sprague-Dawley rats at 1.2 g/Kg. The rats (*n* = 165) were divided randomly into four groups: simple hepatocyte transplantation group (Group A), collagen and hepatocyte transplantation group (Group B), and nanoparticles, collagen, and hepatocyte transplantation group (Group C), and nanoparticles transplantation group (Group D).

### 2.5. Porcine Hepatocyte Culture

Porcine hepatocytes were isolated by an *in situ* recirculating collagenase perfusion method as described previously [8–11]. The yield of hepatocytes was (4.5~5.0) × 10^7^/g. The mean viability of hepatocytes was 97% by trypan blue exclusion. The isolated hepatocytes were suspended in RPIM1640 medium supplemented with 10% NBS, 200 *μ*g/L hydrocortisone, 100 *μ*g/L insulin, 200 *μ*g/L HGF, 100000 U/L penicillin, and 100 *μ*g/L streptomycin. The isolated hepatocytes were divided into three groups and incubated at 5 × 10^6^/mL in 5% CO_2_ atmosphere with 100% humidity at 37°C. Group A: isolated hepatocytes were cultured for 24 hr. In the first 12 hr, the hepatocyte suspensions were agitated for 5 min every 30 min. Then they were centrifugated at 800 rpm and hepatocytes were resuspended in the above medium for 12 hr. 5 mL hepatocyte suspensions with 1 × 10^7^ cells/mL were transplanted into the peritoneal cavity of ALF rats. Group B: isolated hepatocytes were cultured for 12 hr and agitated for 5 min every 30 min. Then they were centrifugated at 800 rpm and hepatocytes were resuspended in the above medium. The hepatocyte suspensions with 4 × 10^7^ cells/mL, collagen type I (0.4% collagen type in 0.1 N acetic acid), 10 × RPMI1640, 100% NBS, and 1 N NaOH were mixed. The volume ratio of the above five components was 2.5 : 5 : 1 : 1 : 0.5. The final collagen concentration was 2 mg/mL, the density of hepatocytes was 1 × 10^7^/mL, and pH of solution was 7.4. The mixture was dropped into 6-well cell culture plates at 5 mL for every well and cultured in 5% CO_2_ incubator at 37°C. Porous gel was formed after 2 to 3 hr. Then 5 mL RPMI1640 medium was added to make the gel suspended in the medium. The hepatocyte suspensions were further cultured in 5% CO_2_ incubator at 37°C. The total culture time after collagen addition was 12 hr. At last, the gel containing hepatocytes was transplanted into the peritoneal cavity of ALF rats. Group C: isolated hepatocytes were cultured with 100 mg/L PLA-O-CMC nanoparticles for 12 hr and agitated for 5 min every 30 min. Then the medium was centrifugated at 800 rpm and hepatocytes were resuspended in the above medium. The hepatocyte suspensions, collagen type I, 10 × RPMI1640, 100% NBS, and 1N NaOH were mixed in the same method as Group B. The final collagen concentration was 2 mg/mL, the density of hepatocytes was 1 × 10^7^/mL, concentration of the nanoparticles was 100 mg/L, and pH of solution was 7.4. The mixture was dropped into 6-ell cell culture plates at 5 mL for every well and cultured in 5% CO_2_ incubator at 37°C. Porous gel was formed after 2-3 hr. Then RPMI1640 medium was added to make the gel suspended in the medium. The hepatocyte suspensions were further cultured in 5% CO_2_ incubator at 37°C. The total culture time after collagen addition was 12 hr. At last, the gel containing hepatocytes was transplanted into the peritoneal cavity of ALF rats.

### 2.6. HTx

HTx was done in Sprague-Dawley rats at 48 hr after D-gal injection. Under ether anesthesia, the abdomen cavity was opened through median incision of abdomen. In Group A, hepatocyte suspensions were injected into the lesser omentum sac of ALF rats. In Groups B and C, gel containing hepatocytes and gel containing hepatocytes cultured with nanoparticle were transplanted into the peritoneal cavity of ALF rats and wrapped up with greater omentum, respectively. The total number of transplanted hepatocytes was 5 × 10^7^. In Group D, 100 mg/L PLA-O-CMC nanoparticles were injected into the lesser omentum sac of ALF rats. Abdomen wall was sutured in layer. After transplantation, the rats in three groups were raised in different cages and drank water with 10% glucose. No immunosuppressive reagents were administered throughout the experiment.

### 2.7. Determination of Plasma HGF and VEGF Levels

Plasma HGF level was determined in batches of five rats each before HTx and at 2, 6, 12, 24, 36, and 48 hr after HTx. Plasma VEGF level was determined in batches of five rats each before HTx and at 48, 72, 84, and 96 hr after HTx. Blood sample was collected from abdominal aorta of rats and put into the test tube with ethylene diamine tetraacetic acid (EDTA). The samples were centrifuged at 3000 rpm for 10 min and stored at −80°C until growth factor assays were performed. The levels of plasma HGF and VEGF were analyzed by an enzyme-linked immunosorbent assay (kits were supplied by Fanbang Co, Dalian, China), according to the manufacturer's protocols. All samples were tested in duplicate. The optical density was read within 30 min using enzyme mark meter set to wavelength of 450 nm and 630 nm. The levels of HGF and VEGF were calculated from a standard curve.

### 2.8. Determination of Liver Functions

Changes of albumen (ALB), alanine aminotransferase (ALT), total bilirubin (TB), and NH_3_ levels in the plasma were determined with an automatic biochemical analyzer (7600-020, Hitachi, Japan).

### 2.9. Determination of Kiel 67 Antigen Labeling Index (Ki-67 LI)

*Ki-67 LI* was evaluated. Immunochemistry technique was used. The paraffin sections of recipients' livers were stained with hematoxylin. Under light microscope, 5 visual fields were randomly selected, and the number of cells with buffy nucleus among 1000 cells per visual field was counted, and then the percentage of cells with buffy nucleus was calculated [12].

### 2.10. Statistical Analyses

All results were expressed as mean ± standard deviation. Statistical analyses were performed using Stata 7.0 software. Statistical significance was determined by analysis of variance (ANOVA) with Student's *t*-test. A *P* value of less than .05 was considered statistically significant.

## 3. Results

### 3.1. Plasma HGF Levels

**Figure 1:**
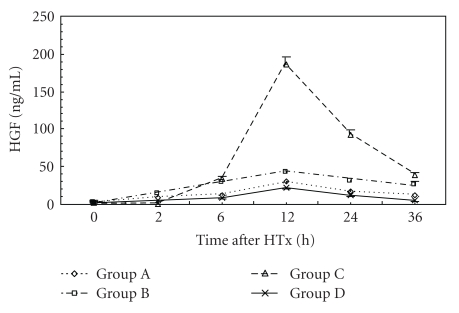
Time course of changes in plasma HGF levels in three groups. Error bars indicate standard deviations (*n* = 5). Statistical differences were determined by ANOVA using Stata 7.0 software.

Plasma HGF levels were indetectable in normal rats. They were increased at 48 hr after D-Gal injection. They continued to elevate after HTx with a distinct peak at 12 hr. They gradually declined thereafter (Figure 1). The rats in Group C showed highest plasma HGF levels at 2, 6, 12, 24, and 36 hr after HTx and lowest HGF level at 48 hr compared with other groups (*P* < .05). Plasma HGF levels at 2, 6, 12, 24, and 36 hr after HTx in Group B were higher than other groups (*P* < .05).

### 3.2. Plasma VEGF Levels

**Figure 2:**
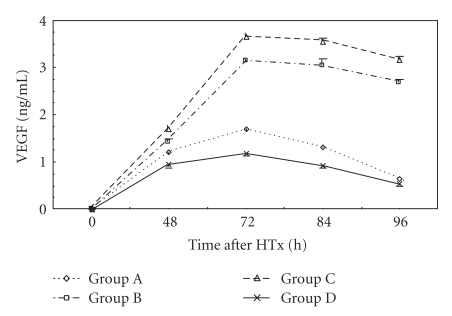
Time course of changes in plasma VEGF levels in three groups. Error bars indicate standard deviations (*n* = 5). Statistical differences were determined by ANOVA using Stata 7.0 software.

Plasma VEGF levels were indetectable at 48 hr after D-Gal injection. They were increased at 48 hr after HTx with a distinct peak at 72 hr. They gradually declined thereafter (Figure 2). The rats in Group C showed highest plasma VEGF levels at 48, 72, 84, and 96 hr after HTx compared with other groups (*P* < .05). Plasma VEGF levels at 48, 72, 84, and 96 hr after HTx in Group B were higher than other groups (*P* < .05).

### 3.3. Liver Functions

**Figure 3:**
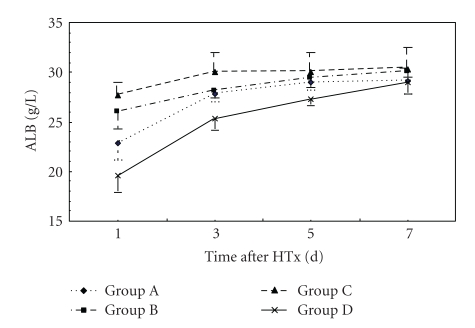
Time course of changes in plasma ALB levels in three groups. Error bars indicate standard deviations (*n* = 5). Statistical differences were determined by ANOVA using Stata 7.0 software.

**Figure 4:**
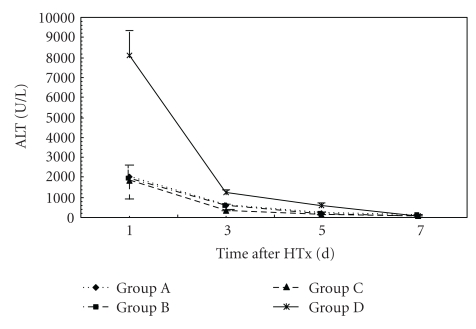
Time course of changes in plasma ALT levels in three groups. Error bars indicate standard deviations (*n* = 5). Statistical differences were determined by ANOVA using Stata 7.0 software.

**Figure 5:**
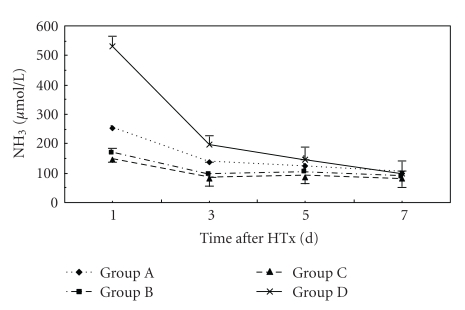
Time course of changes in plasma NH_3_ levels in three groups. Error bars indicate standard deviations (*n* = 5). Statistical differences were determined by ANOVA using Stata 7.0 software.

**Figure 6:**
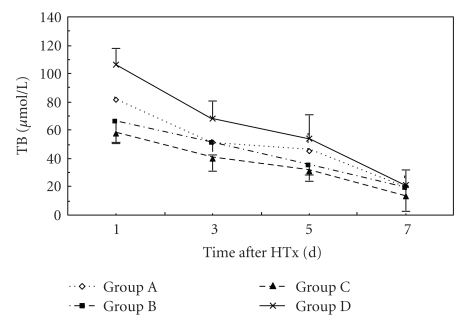
Time course of changes in plasma TB levels in three groups. Error bars indicate standard deviations (*n* = 5). Statistical differences were determined by ANOVA using Stata 7.0 software.

At 24 hr after HTx, ALB level in Group C was higher than that in Groups A and D (*P* < .05). NH_3_ level in groups B and C was lower than that in Groups A and D (*P* < .05). At 72 hr after HTx, ALT and NH_3_ levels in Group C were lower than those in Groups A and D (*P* < .05). ALB level in Group C was higher than that in Groups A and D (*P* < .05). ALT level in Group C was lower than that in other groups. There was no significance in ALB, ALT, TB, and NH_3_ levels between Groups A and B. At days 5 and 7 after HTx, there were no significance in ALB, ALT, TB, and NH_3_ levels in all groups (Figures 3, 4, 5, and 6).

### 3.4. Ki-67 LI in Hepatic Tissue

**Figure 7:**
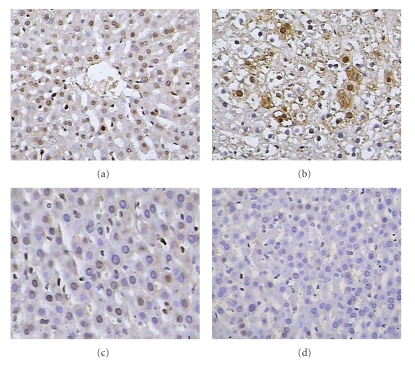
Liver Ki-67 LI of ALF rats at 5d after HTx (×400).

**Figure 8:**
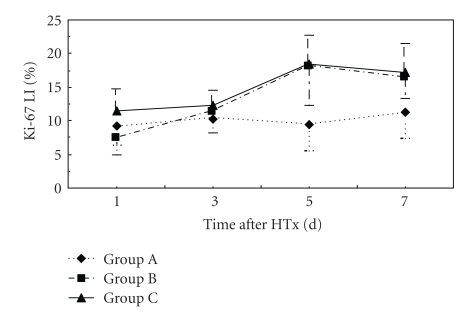
Time course of changes in liver Ki-67 LI in three groups. Error bars indicate standard deviations (*n* = 5). Statistical differences were determined by ANOVA using Stata 7.0 software.

Figure 7 showed pathology of livers of ALF rat at 5d after HTx under light microscopy. Compared with Group B, Group C had significant high Ki-67 LI at day 1 after HTx (*P* < .05). Ki-67 LI in Groups B and C was higher than that in Groups A and D at days 5 and 7 after HTx (*P* < .05) (Figure 8).

## 4. Discussion

ALF is associated with a high mortality. Patient survival depends in part on the regenerative capacity of the remaining hepatocytes. Orthotopic liver transplantation has emerged as an effective treatment for ALF [13]. However, wide application of this therapeutic modality is limited primarily by lack of donors, inability to procure organs on short notice, and high cost. Making a decision about transplantation depends on whether sufficient liver regeneration can occur before the onset of irreversible complications of liver failure. 

HTx can provide opportunity of liver regeneration for ALF patients by liver function support. Liver regeneration is a complex course in which many factors participate in regulation [14]. The process of liver regeneration mainly included three key stages. Start stage: hepatocytes in phase G0 entered phase G1 under the regulation of TNF-*α*, IL-6, and growth factors. Progress stage: hepatocytes in phase G1 entered phase S under the regulation of cyclin-dependent kinase system, HGF, and TGF-*α*. Termination stage: growth of hepatocytes stopped under the regulation of TGF-*β* and nandrolone phenylpropionate [15, 16]. 

It was showed that the hepatic parenchymal cells in phase G0 could be activated after 30 min of hepatic injury and entered the cell proliferation cycle. Their DNA synthesis arrived at peak at 24 hr. Their proliferation was completed on the whole at 72 hr. Then the structural and functional reconstruction of liver began. After 7 to 10 days, the liver recovered both in the volume and weight [17]. 

HGF has been isolated and purified from the plasma of patients with fulminant hepatic failure and from rat platelets. It is a heterodimeric molecule composed of a 69-kD alpha chain and a 34-kD beta chain. HGF is produced by hepatic mesenchymal cells such as lipocyte (Ito cells), Kupffer cells, and sinusoidal endothelial cells (SEC). HGF is considered to be important in the stimulation of DNA synthesis of hepatocytes. HGF produced in nonparenchymal liver cells acts on injured parenchymal hepatocytes through a paracrine mechanism via the c-met tyrosine kinase receptor in the surface of cellular membrane. It promoted liver regeneration by enhancing mitosis of hepatocytes, inhibiting apoptosis of hepatocytes, promoting the recovery and reconstruction of morphology of liver tissue, and promoting growth of blood vessel endothelium and formation of capillary. Elevated serum HGF levels have been reported after partial liver resection and in the settings of ALF [18, 19]. Arkadopoulos et al. [20] induced ALF rat model by total liver resection after transplanting hepatocytes into spleen. They found that plasma HGF increased at 12 hr after operation. In this study, we found that plasma HGF levels were increased at 48 hr after D-Gal injection, which was consistent with other reports.

Recent studies have shown that VEGF, a most potent angiogenic factor, plays an essential role in liver regeneration. Exogenous VEGF administration is able to stimulate liver regeneration following acute severe liver injury and partial hepatectomy in rats [21]. It has also shown that the serum VEGF levels in the survivors of ALF significantly increased in the recovery phases compared with corresponding levels on admission, suggesting that VEGF plays an important role in liver regeneration after ALF. Namisaki et al. [22] has shown that HGF is a potent inducer of VEGF secretion by HepG2 cells. Liver SEC proliferation is induced by VEGF during liver regeneration. Shimizu et al. [23] found that proliferation of liver SECs followed hepatocyte proliferation by 24 to 48 hr. This is probably related to formation of new vasculature to supply blood to the regenerated tissue. The authors suggested that induction of VEGF secretion by hepatocytes may constitute a pathway, whereby HGF originating from either nonparenchymal liver cells or distant organs not only stimulates hepatocyte proliferation but also mediates liver SEC proliferation and survival as an indirect angiogenic effect. In addition, VEGF produced by hepatocytes may stimulate liver SEC to produce growth factors, including HGF, with liver protective/proliferative effects. Thus, VEGF and HGF appear to have complementary roles in liver injury and regeneration.

It has been shown that the VEGF expression increased markedly during liver regeneration induced either by partial hepatectomy or drug intoxication [23]. Akiyoshi et al. [24] found that VEGF level correlated with Child-Pugh class of liver function. The lower the Child-Pugh class was, the lower the level of VEGF was. In our study, plasma VEGF levels in every group were increased at 48 hr after HTx with a peak at 72 hr. Liver regeneration involves in the reconstitution of hepatic sinusoids. VEGF promoted proliferation of SEC and hepatocytes and reconstitution of hepatic sinusoids [22]. VEGF produced effect through combination with high affinity receptor, KDR/Flk-1, that is expressed almost exclusively on the surface of SECs [25]. It was found that VEGF expressed predominantly in periportal hepatocytes at 48 to 72 hr after partial hepatectomy. Gupta et al. [26] transplanted the hepatocytes into the spleen of inbreeding line, F344 rats and found that expression of VEGF could be detected when the transplanted hepatocytes entered the hepatic sinusoids and integrated into hepatic plates through endothelial fenestrations after 8 hr of transplantation. 

Ki-67 antigen, a sensitive indicator of liver regeneration, is involved in DNA synthesis and is closely related to cell proliferation [27, 28]. In our study, all groups showed liver regeneration signs at different degrees and in different times after HTx, which indicated the effect of HTx. The increase of Ki-67 LI appeared earliest in Group A. Compared with Group B, Group C had significant higher Ki-67 LI at day 1 after HTx. Ki-67 LI in Groups B and C was higher than that in Group A at days 5 and 7 after HTx (*P* < .05). We speculated that the increase of Ki-67 LI may be related to the increase of plasma HGF and VEGF levels. Boudjema et al. [29] considered that the main mechanism of raising survival rate of ALF patients included supplying temporary liver function support, promoting remnant liver regeneration and recovery of liver function through production of HGF. In this study, HGF levels were elevated before HTx and continued to increase after HTx with a peak at 12 hr. Whether this is caused by impaired HGF clearance, increased synthesis at extrahepatic sites, or both remains to be seen. VEGF level increased at 48 hr after HTx with a peak at 72 hr. The results indicated that elevated blood HGF level switched on liver regeneration. The continuous elevation of blood HGF level promoted liver regeneration further. VEGF played an important role in reconstitution of hepatic sinusoids. Plasma HGF levels were decreased after 12 hr following HTx, which may be related with the increased clearance of HGF.

In this study, plasma HGF levels during 36 hrs after HTx and VEGF levels after HTx in Group C were found to be highest in all groups. Ki-67 LI was highest in Group C at days 5 to 7 after HTx, which indicated the most active liver regeneration. The improvement of liver functions in Group C was most rapid than other groups. It was postulated that better liver regeneration was mainly due to the higher cytoactive porcine hepatocytes cultured with PLA-O-CMC nanoparticles and embedded in collagen gel. These hepatocytes had better effect on liver function substitution and could make livers produce more HGF and VEGF which could promote the regeneration and restoration of injured liver [5]. 

 In summary, we have demonstrated that rats with ALF triggered a regenerative response in the native liver because of elevated plasma HGF levels after D-Gal injection and continuous increase of HGF after HTx. Elevated plasma VEGF after HTx was helpful in reconstitution of hepatic sinusoids. Transplantation of porcine hepatocytes cultured with PLA-O-CMC nanoparticles and embedded in collagen gel promotes liver regeneration in ALF rats.
